# Interobserver Agreement of Novel Classification of Central Serous Chorioretinopathy

**DOI:** 10.7759/cureus.25925

**Published:** 2022-06-14

**Authors:** Niroj K Sahoo, Deepika C Parameshwarappa, Mahima Jhingan, Filippo Tatti, Claudio Iovino, Enrico Peiretti

**Affiliations:** 1 Ophthalmology, L V Prasad Eye Institute, Anant Bajaj Retina Institute, Vijayawada, IND; 2 Ophthalmology, Seth Gordhandas Sunderdas Medical College, King Edward Memorial Hospital, Mumbai, IND; 3 Ophthalmology, University of Cagliari, Cagliari, ITA; 4 Ophthalmology, University of Campania Luigi Vanvitelli, Naples, ITA

**Keywords:** kappa, interobserver agreement, validation, classification, central serous chorioretinopathy, cscr

## Abstract

Objective

To validate the newly proposed multimodal-imaging-based classification for central serous chorioretinopathy (CSCR).

Methods

This was a retrospective study performed in a total of 87 eyes of 44 patients with a diagnosis of CSCR. Multimodal images in the form of auto-fluorescence, fundus fluorescein angiography, indocyanine green angiography, and optical coherence tomography (OCT) images, of all the patients, were presented to two masked retina specialists. The masked observers graded each eye into simple or complex; primary, recurrent, resolved; and specific features such as foveal involvement, outer retinal atrophy, and choroidal neovascularization (CNV). Interobserver agreement was assessed using Cohen's kappa. In areas of non-consensus, a detailed discussion was carried out with a third independent grader.

Results

The mean age of the 44 patients (32 males and 12 females) was 49.2±9.3 years. We found a moderate-strong agreement between the two observers in all subclassifications, that included “simple or complex” (kappa value=0.91, 95% CI 0.82-0.99, p<0.001); “primary/recurrent/resolved” (kappa value=0.88, 95% CI 0.80-0.96, p<0.001) and “foveal involvement” (kappa value=0.89,95%CI 0.8-0.98, p<0.001). However, there was less agreement between the two graders with respect to classification of “outer retinal atrophy” (kappa value=0.72, 95%CI 0.57-0.87, p<0.001) and “presence/absence of CNV” (kappa value=0.75, 95% CI 0.58-0.92, p<0.001). Non-consensus in categorizing "outer retinal atrophy" was seen in eyes with sub-retinal hyper-reflective material (SHRM) and outer nuclear layer (ONL) thinning overlying subretinal fluid, and non-consensus in categorizing "CNV" was seen in eyes with inner choroidal atrophy.

Conclusion

Our study reports the validity and strong interobserver agreement in several aspects of the multimodal-imaging-based classification. This could support its implementation in clinical practice and pave way for appropriate treatment guidelines.

## Introduction

Central serous chorioretinopathy (CSCR) is one of the most enigmatic diseases of the choroid. While most of the cases are self-resolving, a considerable percentage of individuals manifest a chronic, recurring, and relapsing course [1-4]. The long-standing nature of the disease often results in permanent damage to the outer retina, retinal pigment epithelium (RPE), and choroid, leading to irreversible vision loss [1,5]. Thus, early identification and treatment play a pivotal role in the management of such cases. However, to do this, a well-defined classification is required that could give us information about the disease characteristic and treatment guidelines.

Traditionally, CSCR has been classified as acute or chronic CSCR based on the duration of sub-retinal fluid (SRF). The term "acute CSCR" is reserved for eyes with SRF accumulation of less than four months, while the term "chronic CSCR" is applied for eyes with more than four months duration [1,2,5]. However, this classification is dependent on the patient’s anamnesis and the cut-off duration is often loosely used in various publications, ranging from three to six months, resulting in variability in nomenclature among ophthalmologists [6]. Also, eyes diagnosed with acute CSCR often encounter multiple recurrences and relapses resulting in clinical findings often overlapping with that of chronic CSCR. This shortcoming was dealt with to some extent by Daruich et al., where the authors sub-classified CSCR into persistent CSCR (SRF lasting more than four months); recurrent CSCR (new SRF after a period of inactivity), inactive CSCR (no current disease activity) and chronic CSCR (widespread RPE decompensation) [5]. Though it did introduce uniformity in the nomenclature among ophthalmologists, it did not address the overlapping clinical characteristics in some of the cases of recurrent CSCR and chronic CSCR cases, which can result in confusion in classification, especially in situations with improper history. There was an unmet need for a newer classification that can characterize the disease based on the clinical characteristic at a given time point. Recently, Chhablani et al. proposed a novel classification based on the multimodal imaging characteristics with further clarity into severity and extent [7]. The classification included parameters such as area of RPE abnormalities, characteristics of SRF (primary/recurrent/resolved), persistence (more than six months), outer retinal atrophy, CNV, and foveal involvement. In this study, we aim to validate this new classification using multimodal images and address a few challenges and controversies.

## Materials and methods

This was a retrospective, observational study performed using data retrieved from two tertiary eye care institutions (India and Italy). The study adhered to the tenets of the declaration of Helsinki and was approved by the institutional review board of the LV Prasad Eye Institute, India (LEC BHR-R-12-20-564). Patients having a diagnosis of unilateral or bilateral CSCR, and satisfying the inclusion and exclusion criteria, were consecutively selected. Patients with any co-existing retinal or choroidal conditions like retinal vein occlusions, diabetic retinopathy, retinal/choroidal degenerations, or dystrophies, that influence the autofluorescence and angiographic pattern were excluded from the study. Only patients with a well-documented autofluorescence, optical coherence tomography (OCT) (including scans passing through fovea), and fundus fluorescein angiography images, were included. The images were checked for proper quality, and files with poor-quality images that could possibly impact the grading process were excluded. Data collected from the files included the demographic data, ocular and systemic history, and any history of previous episodes of CSCR and treatment for the same.

Multimodal images of both eyes of each patient were compiled and presented to two masked retina specialists (NKS, DCP). The images were captured using consoles from different manufacturers from the two centres. OCT images were acquired using Heidelberg Spectralis HRA and OCT (Heidelberg Engineering, Heidelberg, Germany), Triton swept-source (SS)-OCT device (Topcon Corporation, Tokyo, Japan), Cirrus HD-OCT (Carl Zeiss Meditec, Dublin, CA). Fundus fluorescein angiography (FFA)/indocyanine green angiography (ICG)/fundus autofluorescence (FAF) images were acquired using Heidelberg Spectralis HRA and OCT (Heidelberg Engineering, Heidelberg, Germany), Zeiss FF 450 plus IR (Carl Zeiss Meditec AG, Jena, Germany). Based on the new classification [7], the graders were asked to classify CSCR initially into either simple (less than two disc diameters RPE alterations) or complex (more than/equal to two disc diameters RPE alterations) CSCR, based on FAF. It was then further classified into primary CSCR (first known episode of SRF based on history and examination), recurrent CSCR (presence of SRF with a history or signs of resolved episodes), and resolved CSCR (absence of SRF on OCT). The term “atypical” was reserved for cases presenting with a bullous variant of CSCR or those with RPE rips. The graders were also asked to grade the disease with respect to other parameters like outer retinal atrophy (yes/no), foveal involvement in the form of SRF, outer retinal atrophy or RPE detachment (yes/no), and presence of CNV (yes/no) [7]. Persistence of activity for more than six months was not taken into consideration given the introduction of possible bias during retrieval of this information from medical records. The observers were asked to grade the type of CSCR, remotely, using an online survey platform (Google forms). The responses under each sub-classification were given a grade and the grades from the two observers were compared. The compiled data were evaluated for areas of non-consensus, following which a detailed discussion with the third reviewer (MJ) was done, and possible causes of non-consensus were discussed.

Statistical analysis was performed using SPSS statistical software, version 20 (SPSS, Inc., Chicago, IL, USA). Continuous variables were reported as mean and standard deviation (SD). Interobserver agreement between the graders was assessed using Cohen's kappa (95% confidence intervals). Kappa values of 1.0-0.91 were considered as almost/near-perfect agreement, 0.80-0.90 as strong agreement, 0.60-0.79 as moderate agreement, 0.40-0.59 as weak agreement, 0.21-0.39 as minimal agreement, and 0-0.20 as no agreement [8]. A p-value of less than 0.05 was taken as statistically significant.

## Results

**Figure 1:**
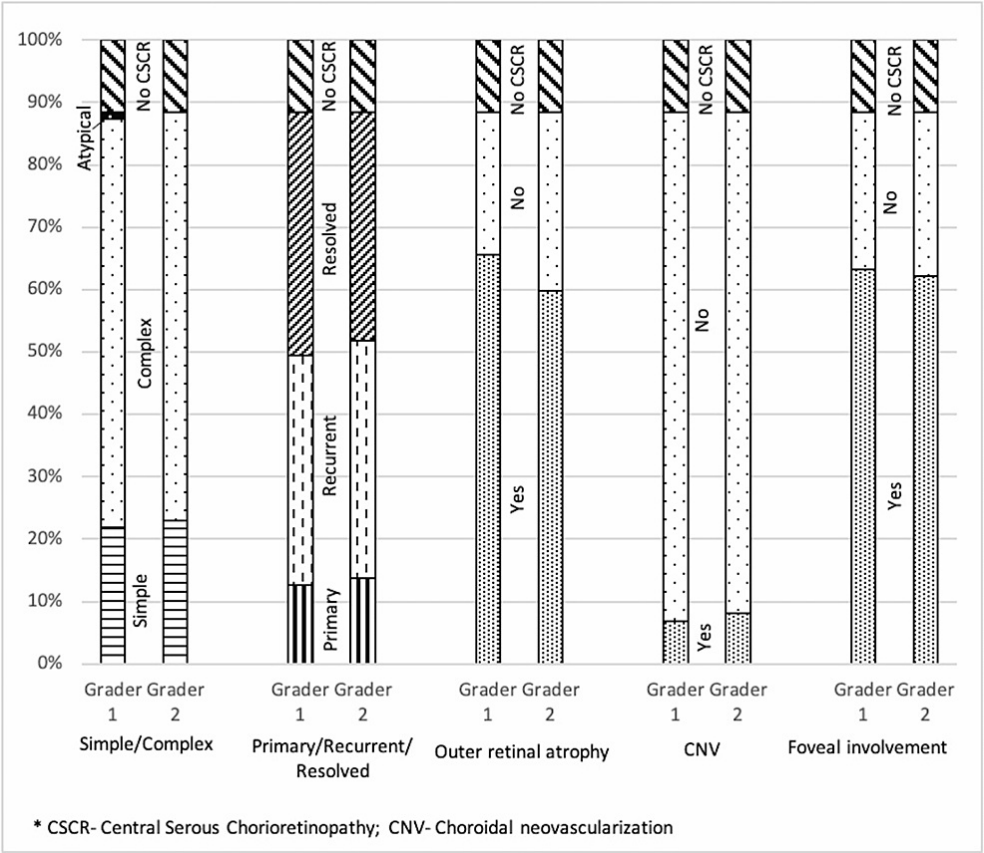
Chart showing a comparison of the number of eyes under each sub-classification

A total of 87 eyes of 44 patients, with unilateral or bilateral CSCR, were graded. One eye of one patient was excluded due to angiographic evidence of old retinal vein occlusion. The mean age of the cohort was 49.2±9.3 years, with 32 males and 12 females. Eight patients had a documented history of previous treatment with either laser, photodynamic therapy, or eplerenone. The number of eyes under each sub-classification graded by both the graders has been compared in Figure 1. Kappa value for the entire classification was 0.89 (95% CI 0.88-0.90). A summary of agreements between the two graders is given in Table 1.

**Table 1:** Table showing agreement levels for each sub-classifications

	Raw percentage of agreement	Kappa (95% CI)	p-value
Area of involvement	95.4%	0.91 (0.82-0.99)	<0.001
Characteristic of sub-retinal fluid (Primary/recurrent/resolved)	91.9%	0.88 (0.80-0.96)	<0.001
Outer retinal atrophy	85.1%	0.72 (0.57-0.87)	<0.001
Choroidal neovascularisation	91.9%	0.75 (0.58-0.92)	<0.001
Foveal involvement	94.3%	0.89 (0.8-0.98)	<0.001

Agreement between simple and complex

We found a near-perfect agreement between simple and complex classification (kappa value=0.91, p<0.001). A total of four cases had a disagreement. Three eyes had a disagreement due to borderline measured areas of RPE alteration or missed areas of multifocal RPE alterations. One eye with a history of bullous CSCR was classified as atypical by one observer while complex by another.

Agreement between primary/recurrent/resolved CSCR

Again a strong agreement was seen in terms of the type of CSCR (kappa value=0.88, p<0.001). Seven eyes had a disagreement. Five were due to improper history, leading to differences in terms of primary versus recurrent. 

Agreement on outer retinal atrophy

A moderate agreement was seen (kappa value=0.72, p<0.001). A total of 13 eyes had a disagreement. The areas of disagreement stemmed mostly from improper visualization of outer retinal structures due to sub-retinal hyper-reflective material (SHRM) and also due to categorizing apparent outer nuclear layer (ONL) thinning as outer retinal atrophy.

Agreement on choroidal neovascularisation

A moderate agreement was seen (kappa value=0.75, p<0.001). A total of seven eyes had a disagreement. The differential responses were mostly seen in eyes with inner choroidal atrophy or non-exudative CNV.

Agreement on foveal involvement

A strong agreement was seen (kappa value=0.89, p<0.001). A total of five eyes had a disagreement. The varied response was mainly seen in eyes with an apparent ONL layer thinning overlying a neurosensory detachment or in eyes with irregularities in the photoreceptor outer segments within a neurosensory detachment.

## Discussion

**Figure 2:**
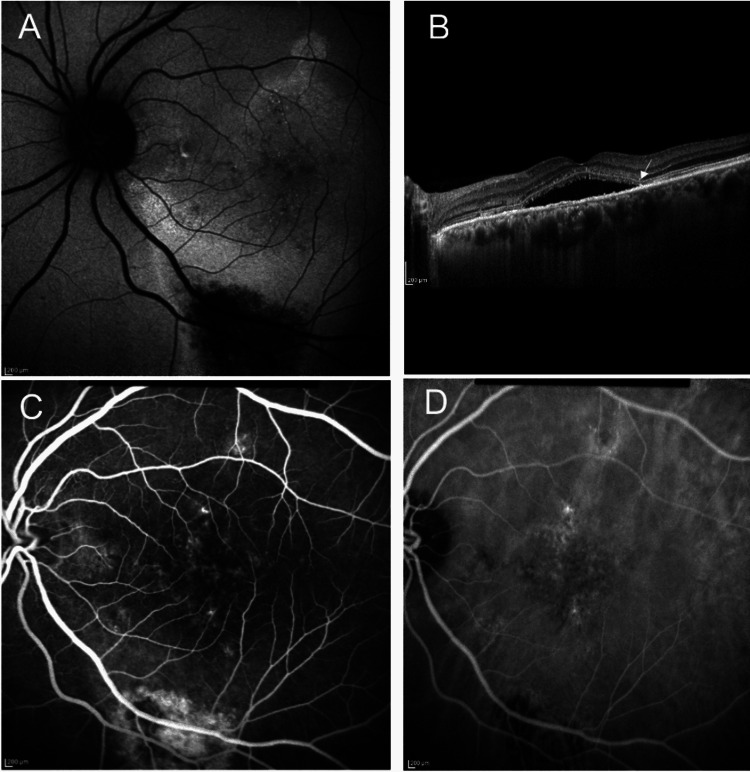
Consensus case example A 61-year-old male presented with decreased vision in both eyes. He had a history of retinal laser treatment 25 years back. On evaluating the left eye, (A) autofluorescence image showed retinal pigment epithelium alteration of the area extending more than two disc diameter; (B) Optical coherence tomography B-scan showed sub-retinal fluid involving fovea along with disruption of ellipsoid zone (arrow); Fluorescein angiography and indocyanine green angiography did not reveal any obvious choroidal neovascularisation (CNV) (C and D). Thus, the case was classified as "complex, recurrent central serous chorioretinopathy, with outer retinal atrophy, without CNV, and with foveal involvement” by both graders.

We found a strong agreement between the two observers in regards to the various sub-sections of the novel CSCR classification. And, this agreement was seen even in images taken from devices of different manufacturers and two different population subsets (Figure 2).

In terms of area of involvement, there were only four situations of disagreement. One of the eyes that had a history of bullous CSCR, was classified as atypical by one grader, while complex by another. Out of the remaining three eyes, two had multifocal areas of subtle RPE changes that were either considered non-related to the CSCR or missed by one of the graders. In the remaining eye, the autofluorescence changes corresponded to the area of SRF and were thus considered as a consequence of sub-retinal deposits by one grader while as a part of RPE abnormality by another. Sub-retinal deposits in CSCR have been seen to show increased autofluorescence and the presence of SRF can lead to a decrease in the autofluorescence in the area [9]. Thus, altered autofluorescence caused by the virtue of the presence of SRF cannot be taken into consideration while calculating the area of RPE alteration.

We also analyzed the disagreement seen with respect to the characteristic of SRF (primary/recurrent/resolved) and found seven eyes with disagreement. Five of the discordance were due to improper history, leading to differences in terms of primary versus recurrent. Although we agree that this is one of the limitations of this sub-classification (as it again depends on the patients’ recollection of episodes and documented history), the classification does include “any clinical signs of previous episodes” in the definition of “recurrent”, which aided in the categorization of a number of cases by the graders (grader’s personal communication) and decreased the bias related to anamnesis. Another aspect of disagreement was with respect to the varied interpretation of SRF pockets and wide areas of ellipsoid zone defects leading to disagreement in two cases as either recurrent or resolved.

**Figure 3:**
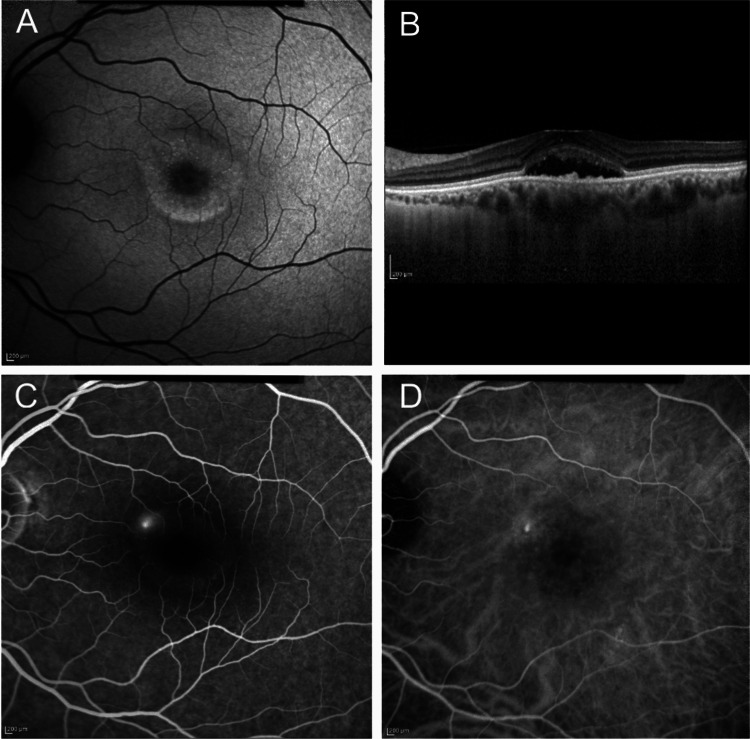
Non-consensus case example A 52-year-old woman presented with a recent onset decrease in vision in the left eye. She had no history of similar episodes or treatment in past. On evaluating the left eye, (A) autofluorescence image showed mottled hyper-fluorescence extending less than two disc diameter in the macular area; (B) Optical coherence tomography B scan showed sub-retinal fluid involving fovea along with elongated photoreceptor outer segments and sub-retinal deposits; (C) Fluorescein angiography did not reveal any obvious choroidal neovascularisation. Both graders classified the case as simple, primary, without choroidal neovascularization, and with foveal involvement. However, one grader graded this case as “with outer retinal atrophy”, while the second grader considered this case as “without outer retinal atrophy”.

On further sub-section analysis, we found a moderate agreement in terms of outer retinal atrophy. A significant number of eyes in our dataset had an SHRM and this can result in difficulty in visualization and differentiation of outer retinal structures like external limiting membrane and ellipsoid zone (Figure 3). These cases can pose a challenge during grading and were one of the reasons for disagreement in this sub-section. Another reason for disagreement was categorizing ONL's apparent thinning as outer retinal atrophy. This again is a controversial aspect of OCT interpretation. For the sake of simplicity, we recommend that ONL thinning outside the area of neurosensory detachment can be taken as outer retinal atrophy and careful analysis needs to be done.

For characterizing the presence or absence of CNV, either an OCT angiography (OCTA) or ICG (if OCTA was not available) was used. Despite this, a moderate degree of agreement was achieved between the graders. The point of disagreement resulted from controversy related to the CNV network versus medium choroidal vessels that are visible in areas of choriocapillaris atrophy. This can be rectified by using OCTA uniformly for all patients, which is more sensitive in detecting CNV networks, and by using proper segmentation of OCTA images [10,11]. Recently, Parameswarappa et al. reported the influence of the presentation of fellow eye findings to the graders, while classifying the type of CSCR [12]. Good reproducibility and agreement were seen in their study, with minimal impact due to the fellow eye status. While the methodology used in their study was similar to ours, the authors analyzed only the "simple" versus "complex" type of CSCR, without including other sub-classifications. 

One of the strengths of our study was that the images were taken from two different population subsets and consoles from different manufacturers. A strong agreement despite this diversity emphasizes the flexibility of this novel classification. Apart from the retrospective study design, one of the major drawbacks of this study was the use of only two graders for analysis. Including more than two graders would have made the study more robust. 

## Conclusions

In this study, a "moderate" to "strong" agreement between graders was seen with respect to all parameters used in the classification. A greater proportion of cases had non-consensus during the assessment of outer retinal atrophy and choroidal neovascularization. Nonetheless, the exclusive image-based criteria for initial classification eliminates bias related to anamnesis and facilitates spot-categorization. This could aid in better acceptance of the classification by practicing ophthalmologists. Future studies can further validate the long-term implications and differences in treatment response after classifying the disease at baseline. 
